# Circulating Methylated DNA to Monitor the Dynamics of RAS Mutation Clearance in Plasma from Metastatic Colorectal Cancer Patients

**DOI:** 10.3390/cancers12123633

**Published:** 2020-12-04

**Authors:** Chiara Nicolazzo, Ludovic Barault, Salvatore Caponnetto, Marco Macagno, Gianluigi De Renzi, Angela Gradilone, Francesca Belardinilli, Enrico Cortesi, Federica Di Nicolantonio, Paola Gazzaniga

**Affiliations:** 1 Liquid Biopsy Unit, Department of Molecular Medicine, Sapienza University of Rome, 00161 Rome, Italy; chiara.nicolazzo@uniroma1.it (C.N.); gia.derenzi@gmail.com (G.D.R.); angela.gradilone@uniroma1.it (A.G.); 2 Department of Oncology, University of Torino, 10060 Candiolo, TO, Italy; ludovic.barault@unito.it (L.B.); federica.dinicolantonio@unito.it (F.D.N.); 3 Candiolo Cancer Institute, FPO-IRCCS, 10060 Candiolo, TO, Italy; marco.macagno@ircc.it; 4 Department of Radiology, Oncology and Pathology, Sapienza University of Rome, 00161 Rome, Italy; salvo.caponnetto@uniroma1.it (S.C.); enrico.cortesi@uniroma1.it (E.C.); 5 Department of Molecular Medicine, Sapienza University of Rome, 00161 Rome, Italy; francesca.belardinilli@uniroma1.it

**Keywords:** circulating methylated DNA, circulating tumor DNA, EGFR inhibitors, liquid biopsy, metastatic colorectal cancer, RAS mutation clearance

## Abstract

**Simple Summary:**

Ongoing clinical trials are recently investigating the efficacy of second-line EGFR inhibitors in initially RAS mutant metastatic colorectal cancer patients who convert to RAS wild-type in plasma, as assessed by circulating tumor DNA (ctDNA) analysis. To this purpose, discriminating between patients with a real clearance of RAS mutations in plasma (real wild-type) from those who do not shed ctDNA is mandatory. The aim of the present study was to confirm the presence of ctDNA in patients with RAS mutation clearance in plasma at different time points, using a colon-cancer-specific five-gene methylation panel. The methylation test confirmed the presence of ctDNA in most RAS wild-type samples at the time of disease progression, thus confirming that the negative selection of RAS mutant clones during the clonal evolution of mutant RAS colorectal cancer is not an infrequent event.

**Abstract:**

The clearance of RAS mutations in plasma circulating tumor DNA (ctDNA) from originally RAS-mutant metastatic colorectal cancer (mCRC) has been recently demonstrated. Clinical trials investigating whether RAS mutant mCRC who “convert” to wild-type in plasma might benefit from EGFR blockade are ongoing. Detection of tumor-specific DNA methylation alterations in ctDNA has been suggested as a specific tool to confirm the tumoral origin of cell-free DNA. We monitored RAS clearance in plasma from patients with RAS-mutant mCRC at baseline (pre-treatment) (T0); after 4 months of first-line therapy (T1); at the time of first (T2) and second (T3) progression. A five-gene methylation panel was used to confirm the presence of ctDNA in samples in which RAS mutation clearance was detected. At T1, ctDNA analysis revealed wild-type RAS status in 83% of samples, all not methylated, suggesting at this time point the lack of ctDNA shedding. At T2, ctDNA analysis revealed wild-type RAS status in 83% of samples, of which 62.5% were found methylated. At T3, 50% of wild-type RAS samples were found methylated. Non-methylated samples were found in patients with lung or brain metastases. This five-gene methylation test might be useful to confirm the presence of ctDNA in RAS wild-type plasma samples.

## 1. Introduction

The use of cell-free DNA (cfDNA) to monitor cancer clonal evolution allows scientists to rapidly identify the occurrence of drug resistance to targeted therapies [1,2,3]. In wild-type (wt) RAS metastatic colorectal cancer (mCRC), the emergence of RAS mutant clones under the selective pressure of epidermal growth factor receptor (EGFR) inhibitors has been widely described, providing a molecular rationale for liquid biopsy-based adaptive therapies [4]. More recently, the negative selection of RAS mutant clones in circulating tumor DNA has been described to occur in some patients with primary RAS mutant mCRC, suggesting for the first time an unpredicted negative selection of RAS mutant clones during the clonal evolution of this cancer type [5]. This observation led to the design of the first clinical trials aimed to investigate the efficacy and safety of second-line EGFR inhibitors plus chemotherapy in initially RAS mutant mCRC patients who convert to RAS wt in plasma at the time of first disease progression (PD) [6,7]. However, it is mandatory to discriminate between patients with an actual clearance of RAS mutations in plasma from those who are negative due to lack in cfDNA of tumor origin. Detection of tumor-specific DNA methylation alterations in circulating tumor DNA (ctDNA) has been suggested as a specific tool to confirm the presence of DNA of tumor origin in plasma [8]. Circulating methylated DNA (cmDNA) defined by a five-gene panel was previously used as a non-invasive treatment-monitoring assay and was associated with outcomes in mCRC patients [9]. The aim of the present study was to monitor the dynamics of RAS mutation clearance in plasma from patients with RAS mutant mCRC early after starting first-line therapy and at the time of progression from first- and second-line treatments. In parallel, a five-gene methylation panel was used to confirm the presence of ctDNA in all plasma samples in which RAS mutation clearance was detected.

## 2. Results

**Figure 1:**
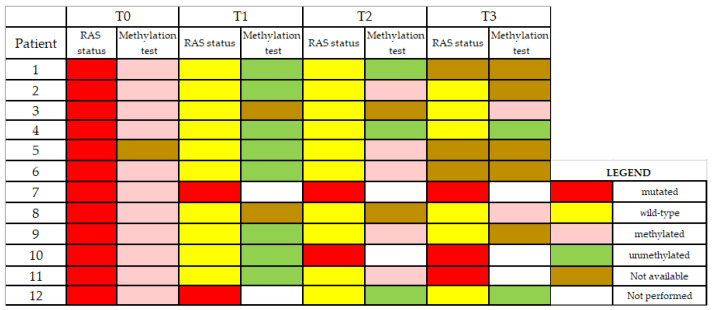
Colorimetric representation of RAS and methylation status in plasma samples from colorectal cancer patients at baseline (T0), after 4 months of first-line therapy (T1), at the time of first progressive disease (PD) (T2), and at the time of second PD (T3).

Twelve patients with RAS mutant mCRC were enrolled, all with evidence of hepatic metastases at the time of diagnosis. The median age was 64.8 (range 47–78). All patients were scheduled to receive first-line therapy. Response to treatment was based on imaging documentation, mostly CT scans, and coded according to Response Evaluation Criteria in Solid Tumors (RECIST) version 1.1. Molecular analysis of primary tumor tissues revealed the following RAS mutations: KRAS G12D (two cases); KRAS G12V (three cases); KRAS G12C (three cases); KRAS G13D (one case); NRAS G12D (one case); NRAS A146T (one case); NRAS Q61R (one case). All formalin-fixed and paraffin-embedded (FFPE) tissue samples were classified as BRAF wt. Characteristics of patient population are shown in Table 1. ctDNA samples were analyzed for specific KRAS and NRAS variants with real-time PCR assays. All plasma samples in which mutant RAS could not be detected at any time points were analyzed for colon-cancer-specific methylation signature, in order to establish the presence of DNA of tumor origin in the circulation (Figure 1). Indeed, the lack of tumor-derived DNA in blood could yield a false negative result in the RAS mutation test and confound the interpretation of RAS clearance.

### 2.1. Plasma ctDNA Analysis at T0

The first plasma ctDNA analysis was performed at baseline (T0), before starting the first-line therapy. Concordance in RAS mutational status between tissue and plasma samples was found for all cases at T0. All plasma samples analyzed at baseline were found methylated.

### 2.2. Plasma ctDNA Analysis at T1

The second plasma sample was collected four months after starting first-line treatment (T1). At that time, ten patients had a partial response (PR) and two had stable disease (SD). ctDNA analysis revealed wt RAS status in 10/12 (83%) patients, while 2/12 (17%) were found RAS mutant, retaining the same mutation as in T0. Methylation analysis of plasma-derived cfDNA (which was available in eight out of the ten plasma samples with wt RAS status at T1) gave negative results in all the samples analyzed, suggesting the lack of ctDNA shedding.

### 2.3. Plasma ctDNA Analysis at First-Line PD (T2)

The third plasma sample was collected at the time of PD from first-line treatment (T2). ctDNA analysis revealed wt RAS status in 10/12 (83%) patients, while 2/12 (17%) were found RAS mutant, one retaining the same mutation as in T0 and T1 and the other one reacquiring the same mutation as in T0, although it found wt RAS at T1. Eight out of the ten wt RAS plasma samples were available for methylation assay. Among the available samples, 5/8 (62.5%) were found methylated, while 3/8 (37.5%) were not. Non-methylated samples were from patients with lung, peritoneum, or brain metastases at the time of PD. In the only patient for whom a tissue biopsy of metastatic site was available (pt. 6), wt RAS was confirmed, in agreement with the results obtained from plasma at the same time point.

### 2.4. Plasma ctDNA Analysis at Second-Line PD (T3)

The fourth plasma sample was available in nine patients at the time of PD from second-line treatment (T3). ctDNA analysis revealed wt RAS status in 6/9 (67%) patients, while 3/9 (33%) were found RAS mutant, with two individuals retaining the same mutation as in T2 and one acquiring a new RAS mutation, different from the one detected at T0. Four out of the six wt RAS plasma samples were available for methylation assay. Among the available samples, 2/4 (50%) were found methylated, while 2/4 (50%) were not. Non-methylated samples were from patients with lung or brain metastases at the time of second-line PD.

## 3. Discussion

According to international guidelines, anti-EGFR therapy is not appropriate for patients with RAS mutant mCRC [10]. In this group of patients, only anti-angiogenic drugs are available as targeted therapy options. Our group has reported for the first time, in RAS mutant mCRC, an unexpected negative selection of RAS mutant clones upon treatment with standard care therapy [6,7]. This indicates that tumor cell population at relapse might be mainly composed of wt RAS clones, possibly opening a therapeutic window with anti-EGFR in patients who “switch” to RAS wt status in plasma at the time of disease progression. We and others recently reported that some RAS mutant mCRC patients who convert to RAS wt status in blood might benefit from EGFR blockade. These preliminary observations lead to the design of the KAIROS trial (eudract n. 2019-001328-36) aimed to evaluate the clinical impact of these findings [11]. Despite the initial skepticism, the clearance of RAS mutation in plasma from RAS mutant mCRC patients has now become a hot topic. To date, different groups are investigating the impact of EGFR inhibitors in patients with RAS mutant tumors at diagnosis who convert to RAS wt disease in blood during therapy [11,12,13,14]. Remarkably, the lack of RAS mutation detection in plasma might also be ascribed to the insufficient amount of ctDNA in the sample. Thus, especially when using a low sensitive ctDNA assay, it is necessary to know how to correctly interpret the wt RAS status as a real switch or, alternatively, as the consequence of lack of ctDNA shedding. Noteworthy, the Idylla ctKRAS Mutation Test is a qPCR assay that has an analytical sensitivity of ≤1% for KRAS mutations in exons 2 and 3 and ≤5% for mutations in exon 4. Compared to ddPCR, Idylla ctKRAS assay might have a reduced accuracy in RAS mutation detection below 1% mutant allele fraction (MAF). In this case, a confirmatory test assessing the presence of ctDNA is necessary.

Some studies have provided evidence that RAS mutations at diagnosis might convert into RAS wt status early in the course of therapies, thus leading to the hypothesis that RAS mutational load reduction might be a surrogate for therapeutic response [15]. Conversely, Klein-Scory et al. recently described RAS conversion in ctDNA early after the first-cycle of chemotherapy, with the maintenance of RAS wt status at the time of progression, assuming that the tumor remained RAS wt through the course of the disease [13]. Authors confirmed the shedding of ctDNA in the course of therapy using a specific methylation test, specifically the presence of methylated WIF1-promoter fragments. Nevertheless, WIF1 methylation test is not tumor specific, being WIF1 methylated in normal colon mucosa as well, suggesting that the use of a broader panel of methylated loci would be more compliant to liquid biopsy analyses to improve the positivity of ctDNA detection [8]. We here sought to monitor RAS wt conversion in ctDNA confirming the presence of ctDNA through a broad five-gene methylation panel. This methylation assay, which has a sensitivity of 0.09% [8] gave positive results in all the plasma baseline samples analyzed. The methylation test was instead found negative in all plasma samples collected after 4 months of first line therapy (6–8 cycles), suggesting that at this time point the RAS wt status was likely to be ascribed to the absence of ctDNA or that RAS mutations were present at level below the limit of detection. This is consistent with the partial response or stability of disease obtained in all patients at that time point. Conversely, at the time of progression from first- and second-line treatments, 62 and 50% of wt RAS plasma samples were found methylated, respectively, suggesting that the wt RAS status in plasma might reflect a wt RAS disease. Of note, only patients with metastatic spread to the peritoneum, lung or brain at T2 and T3 were found negative for methylation test, confirming a tendency of these peculiar metastatic sites to release lower levels of ctDNA [16,17,18]. Although we only analyzed a small patient population, we found a switch to wt RAS disease in a high percentage of patients at the time of disease progression. The main limit of this study is the lack of information concerning RAS status in metastatic site at the time of progressive disease, due to the difficulty to routinely perform biopsies from metastatic sites.

## 4. Materials and Methods

Twelve therapy naïve patients with a primary RAS mutant mCRC at diagnosis (based on tissue mutational analysis) were enrolled prior to first line therapy initiation. FFPE tissue sections from primary tumors were examined by next-generation sequencing according to standard procedures. Blood samples for ctDNA analysis were collected at the following four defined time-points: (1) pretreatment (before starting first-line therapy, T0); (2) after 4 months of first-line therapy (6–8 cycles) (T1); (3) at the time of first PD (T2); (4) at the time of second PD (T3). Blood draws were performed after obtaining informed consent. Authorization to perform liquid biopsies was released by the Regional Ethical Committee (No.:179/16), and the study was conducted in accordance with the Declaration of Helsinki. Plasma samples were obtained by centrifugation of 6 mL of blood at 1500 rpm for 10 min, followed by removal of plasma, which was further centrifuged at 13,000 rpm for 1 min. Plasma samples were stored at −80 °C until Idylla™ (Biocartis) was used to analyze KRAS, NRAS and BRAF mutational status in plasma samples. For methylation analysis, ctDNA was extracted from 1 mL of plasma for each patient using Maxwell RSC system (Promega) according to the manufacturer’s instructions. ctDNA (20 microliters) were subjected to bisulfite conversion using the EZ DNA methylation Gold kit (Zymo Research), with final elution in 40 µl. Bisulfite converted ctDNA was assessed for the methylation status of five genes (EYA4, GRIA4, ITGA4, MAP3K14-AS1, MSC), previously described to be hypermethylated in CRC [9]. The positivity of methylation markers was defined as previously described [9]. All PCR experiments were performed in duplicate in two technical replicates. 

## 5. Conclusions

Recent literature shows that some RAS mutant mCRC switch to wt RAS disease in plasma, and three clinical trials aimed to investigate whether these patients might benefit from EGFR inhibitors are currently ongoing [7,12,16]. In order to avoid false negative results due to the low analytic sensitivity of some liquid biopsy assays, confirming the presence of ctDNA in all wt RAS plasma samples is mandatory [19,20]. We here suggest that the use of a tumor specific, five-gene methylation panel might be helpful to discriminate cases with a real negative clonal selection of RAS mutant clones from those characterized by a lack of ctDNA shedding.

## Figures and Tables

**Table 1:** Clinicopathologic characteristics of patients.

Patient	Age	Gender	Primary Location	Tissue RAS Status	1st Line Therapy	Metastatic Site at 1st PD	2nd Line Therapy	Metastatic Site at 2nd PD
1	68	M	Right	KRAS G12D	Folfoxiri/Bev	Lung/Peritoneum	TAS-102	N.A. †
2	66	M	Left	KRAS G12V	Folfiri/Bev	Liver/Nodes	Folfox/Bev	Liver/Lung
3	76	F	Rectum	KRAS G12V	Folfox/Bev	Liver/Nodes	Folfiri/Bev	Liver/Lung
4	58	F	Right	KRAS G12C	Folfoxiri/Bev	Lung	TAS-102	Brain
5	65	F	Right	NRAS G12D	Folfiri/Bev	Liver	Folfox/Bev	N.A. †
6	78	M	Left	KRAS G13D	Folfiri/Bev	Liver */Lung	Folfox/Bev	N.A. †
7	62	F	Rectum	KRAS G12V	Folfox/Bev	Liver/Lung	Folfiri/Bev	Liver/Lung
8	47	M	Rectum	NRAS A146T	Folfoxiri/Bev	Lung	Folfiri/Aflibercept	Liver/Lung
9	76	M	Left	NRAS Q61R	Folfoxiri/Bev	Liver/Nodes	Folfiri/Aflibercept	Lung
10	58	M	Right	KRAS G12C	Folfox/Bev	Liver/Lung	Folfiri/Bev	Lung
11	64	M	Left	KRAS G12C	Folfiri/Bev	Liver/Lung	Folfiri/Aflibercept	Lung/Peritoneum
12	60	F	Right	KRAS G12D	Folfox/Bev	Brain/Lung	Folfiri/Bev	Brain/Lung

* RAS wild-type status confirmed in metastatic tissue. Bev: bevacizumab F: female; M: male; N.A.: not available; PD: progressive disease; †: deceased.
